# Clinical and Evolutive Features of Tuberculous Meningitis in an Immunosuppressed Adolescent During the COVID 19 Pandemic

**DOI:** 10.3390/biomedicines13071721

**Published:** 2025-07-14

**Authors:** Dalia Dop, Vlad Pădureanu, Rodica Pădureanu, Iulia Rahela Marcu, Suzana Măceș, Anca Emanuela Mușetescu, Ștefan Adrian Niculescu, Carmen Elena Niculescu

**Affiliations:** 1 Department of Pediatrics, University of Medicine and Pharmacy of Craiova, 200349 Craiova, Romania; dalia.dop@umfcv.ro (D.D.); carmen.niculescu@umfcv.ro (C.E.N.); 2 Department of Internal Medicine, University of Medicine and Pharmacy of Craiova, 200349 Craiova, Romania; 3 Department of Physical and Rehabilitation Medicine, University of Medicine and Pharmacy of Craiova, 200349 Craiova, Romania; rahela.marcu@umfcv.ro; 4 Department of Radiology, University of Medicine and Pharmacy of Craiova, 200349 Craiova, Romania; suzana.maces@umfcv.ro; 5 Department of Rheumatology, University of Medicine and Pharmacy of Craiova, 200349 Craiova, Romania; anca.musetescu@umfcv.ro; 6 Department of Orthopedics, University of Medicine and Pharmacy Craiova, 200349 Craiova, Romania; stefan.niculescu@umfcv.ro

**Keywords:** adolescent, tuberculous meningitis, systemic lupus erythematosus, COVID-19

## Abstract

**Background/Objectives:** Tuberculous meningitis is the most severe form of tuberculosis in children, with a high mortality and morbidity rate if it is not diagnosed and treated in a timely manner. The aim of this study is to highlight the challenges associated with establishing a diagnosis of tuberculous meningitis in a child with immunosuppression, given the presence of nonspecific clinical manifestations. **Methods:** We present the case of a 15-year-old adolescent with systemic lupus erythematosus, on immunosuppressive therapy, who is diagnosed with tuberculous meningoencephalitis presenting the clinical, diagnostic and imaging characteristics, as well as the diagnostic traps and limitations associated with this condition. Antituberculosis therapy was started empirically, because there was no improvement in the clinical status with conventional antibiotic therapy; the diagnosis was established 7 days after the start of the antituberculosis treatment, with the help of an acid-fast bacilli culture from the cerebrospinal fluid. **Results:** The course of the tuberculous meningoencephalitis was slowly favorable, despite the superimposed COVID-19 infection. Delay in administering immunosuppressive therapy led to the onset of renal and joint manifestations. **Conclusions:** Tuberculous meningitis is a highly lethal, often underdiagnosed disease with nonspecific clinical and imaging manifestations, which can have a favorable outcome if the diagnosis is established early on and treatment is started promptly.

## 1. Introduction

Tuberculosis meningitis is the leading cause of mortality and morbidity in children with tuberculosis and it is a real public health problem [1]. Due to its atypical onset, nonspecific clinical manifestations, and limited diagnostic tests, most children are diagnosed late, approximately 20% die [2] and many of those who survive have neurological sequelae with long-term negative consequences for the children and their families [3].

The *Mycobacterium tuberculosis* bacillus reaches the pulmonary alveoli through aerosol particles carrying bacilli, binds to specific receptors on the surface of the epithelium and activates specific cells (dendritic, alveolar macrophages or neutrophils) [4], leading to the release of cytokines and chemokines with a role in immune defense [5]. When the immune defense is overcome, the tuberculosis infection occurs, and it manifests through the appearance of tuberculoma in the lung parenchyma or at the meningeal level [6].

*Mycobacterium tuberculosis* crosses the blood–brain barrier through virulence factors that interact with extracellular factors of the brain endothelium to facilitate adhesion of the bacillary endothelium [5], through the “Trojan horse” mechanism, via infected macrophages and neutrophils or by actin rearrangement. Once they enter the brain, they activate microglial cells and astrocytes, triggering a T-cell-mediated inflammatory cascade, including the induction of pro- and anti-inflammatory cytokines, with consequent formation of exudate coating the arteries and nerves, development of vasculitis and disruption of the cerebrospinal fluid flow [7,8].

Patients with systemic lupus erythematosus are at increased risk of developing TB infection because of the immune dysfunction and the immunosuppressive treatment [9]. The manifestations of TB are often atypical in patients with Systemic lupus erythematosus (SLE) because of the aberrant immune response, and some manifestations may mimic the active disease, thus posing a great challenge [10]. Also, because of the immunosuppressive treatment, patients with SLE show non-responsiveness to a purified protein derivative (PPD) skin test and this may lead to the dissemination of TB [11]. Thus, early diagnosis and prompt initiation of treatment are essential in reducing mortality and improving the quality of life of these patients.

## 2. Case Presentation

**Figure 1:**
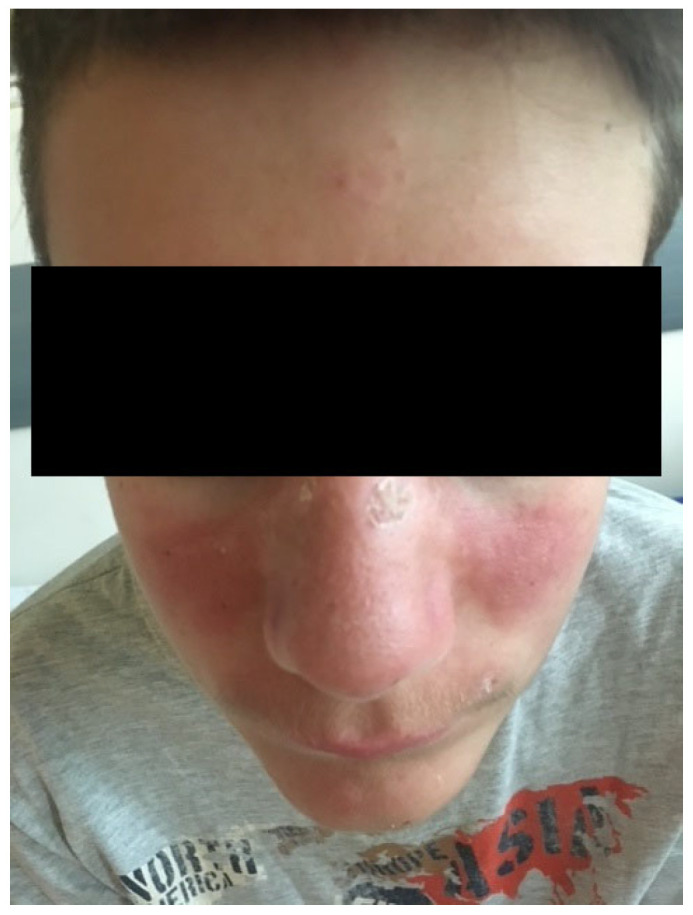
Facial erythema on the malar prominences and the nasal pyramids (butterfly appearance).

**Figure 2:**
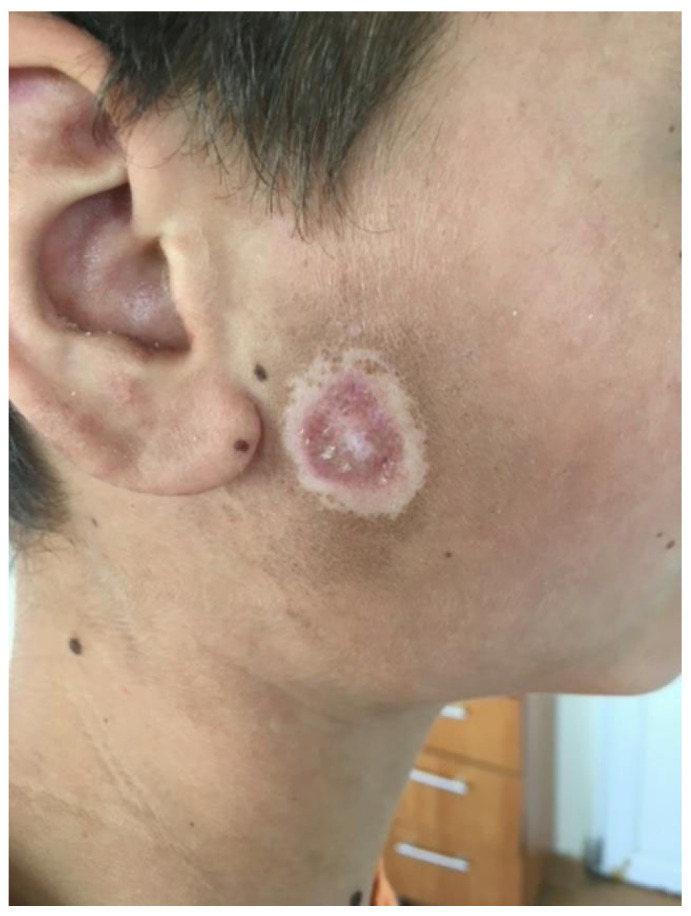
Ulcerations covered by keratotic crusts on the right cheek.

**Figure 3:**
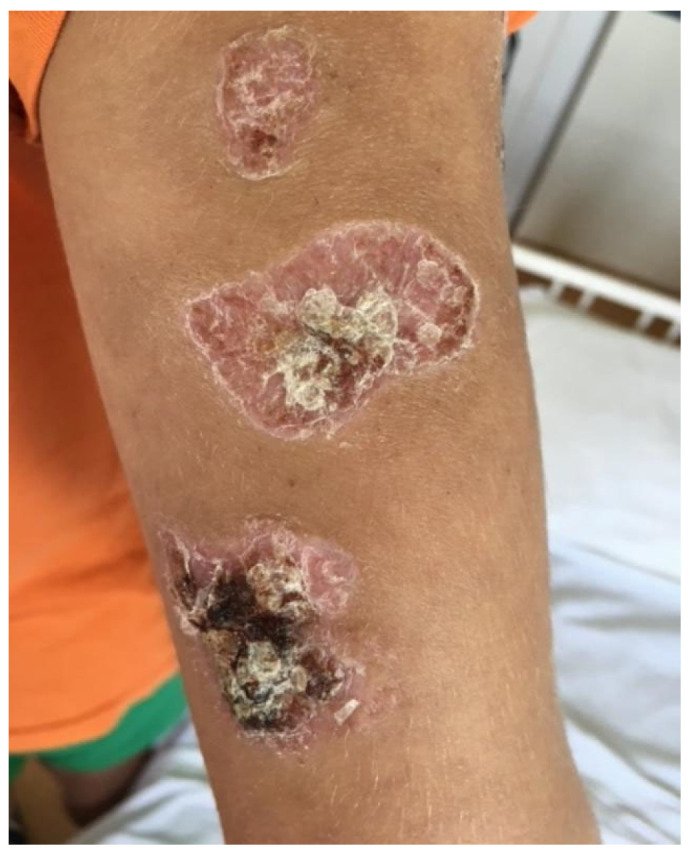
Ulcerations covered by keratotic crusts on both arms, 2/2 cm in size, above the epicondyle.

**Figure 4:**
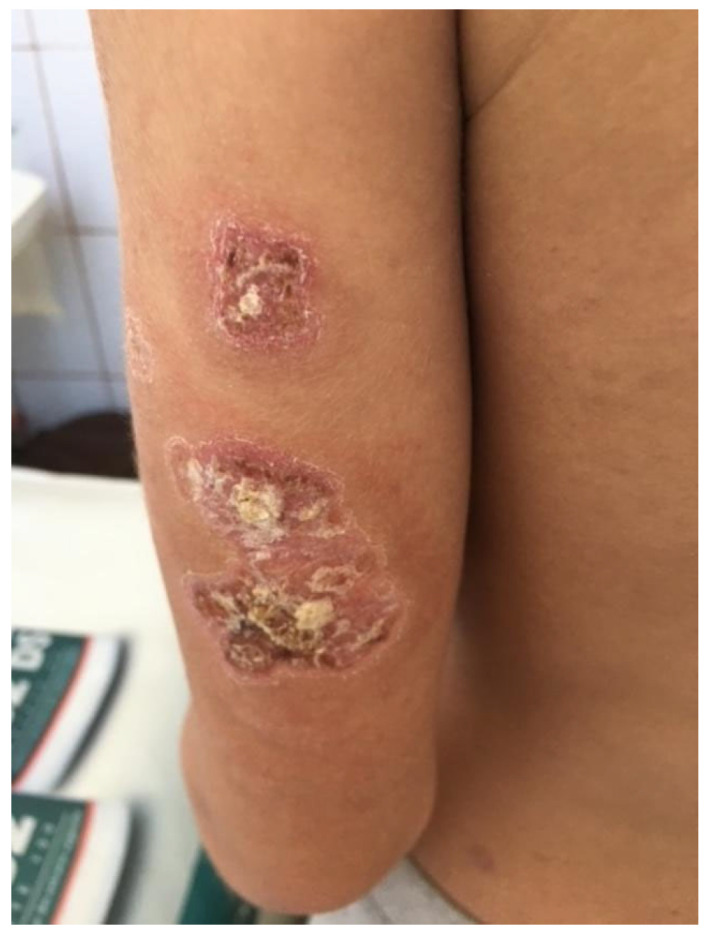
Ulcerations covered by keratotic crusts on both arms, 2/2 cm in size.

**Figure 5:**
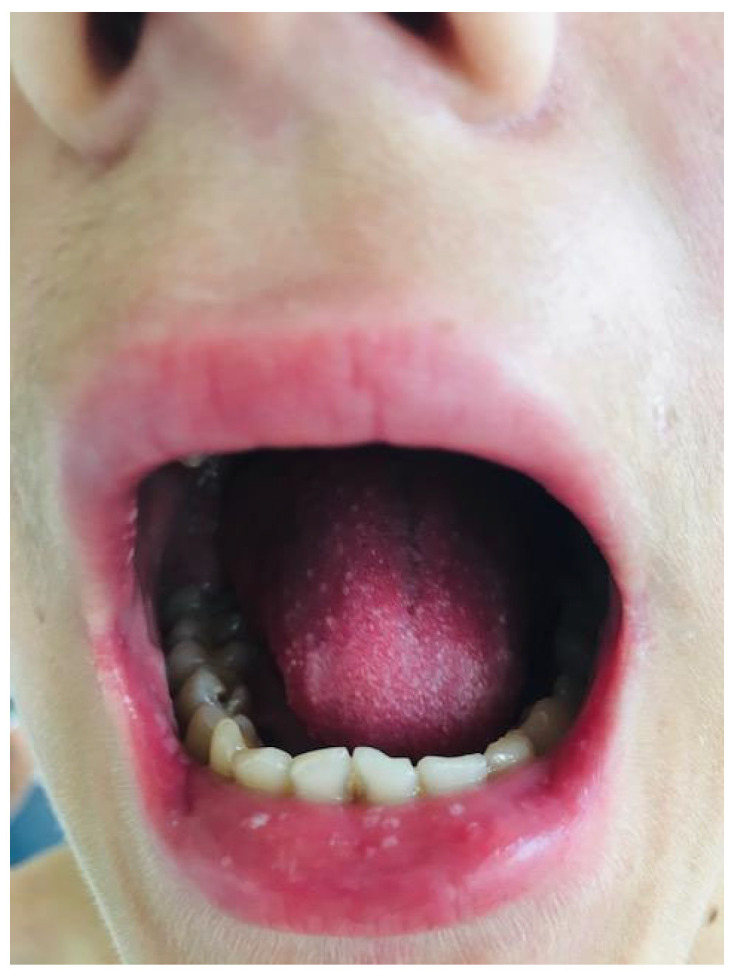
Mouth ulcers, including the upper lip.

We present the case of a 15-year-old adolescent who was hospitalized in our clinic in June 2021. He came in with ulcerative lesions covered by keratotic crusts on both arms and on the face, which had appeared 3 years before and for which he had had intermittent treatment with oral methylprednisolone. The clinical examination showed BMI of 14.9, facial erythema on the malar prominences and the nasal pyramids (butterfly appearance), ulcerations covered by keratotic crusts on the right cheek, on the angle of the mandible and on both arms, 3/2.5 cm in size, above the epicondyle, erythematous papular elements on the upper lip, erythematous papular elements on the cleavage area and upper limbs—on the hands, in sun-exposed areas, bilateral, mobile, painless laterocervical adenopathies, kyphoscoliosis, petechial rush on the hard palate, mouth ulcers, congestive oropharynx, hypertrophic, and hyperemic tonsils (Figure 1, Figure 2, Figure 3, Figure 4 and Figure 5).

The patient presented anemia (hemoglobin—9.9 g/dL) with a reticulocyte count of 0.4% and a negative direct Coombs test, a white blood cell (WBC) count of 2.64 × 10^9^/L, platelet (PLT) level of 58 × 10^9^/L, mildly elevated transaminases (serum glutamic-oxaloacetic transaminase—75 U/L, serum glutamic-pyruvic transaminase—64 U/L), high erythrocyte sedimentation rate (83 mm/h); the C-reactive protein (CRP) level was 22.67 mg/L, low complement C3 (29 mg/dL) and C4 (70 mg/dL), fibrinogen was 251.7 mg/dL, positive fibrin degradation products, serum iron level: 27 µg/dL; transferrin: 1.82 g/L and serum ferritin—1170 ng/mL.

The examinations performed (skin biopsy, lupus cells present, positive antibodies: ANA, AND-ds antibodies, nucleosome) established the diagnosis of systemic lupus erythematosus and treatment with hydroxychloroquine sulphate and low-dose cortisone was initiated. Before beginning treatment, a negative Mantoux skin test was performed; the chest radiography was normal, and HIV infection and acute hepatitis A, B, and C were excluded.

**Figure 6:**
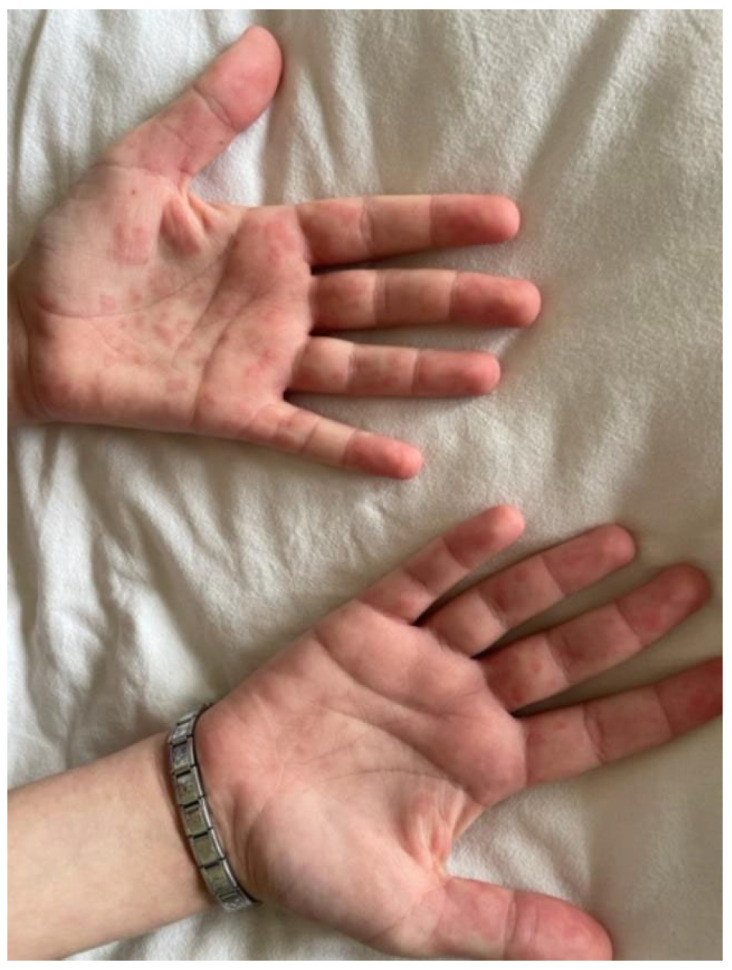
Petechial rashes on his hands and feet.

**Figure 7:**
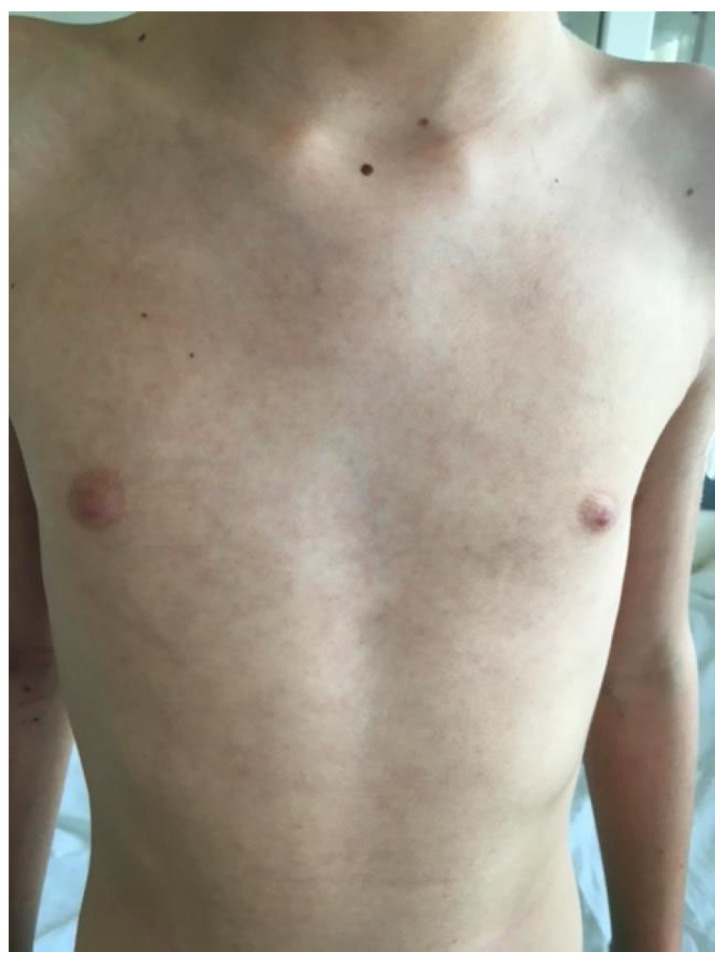
Livedo reticularis on the trunk.

The patient was readmitted after 1 month for high fever, dizziness, asthenia, petechial rashes on his hands and feet, livedo reticularis on the trunk (Figure 6 and Figure 7), and loss of appetite that had started 2 days before. The tests performed showed normochromic anemia (hemoglobin—9.1 g/dL), leucopenia (2.82 × 10^9^/L), elevated acute phase reactants (the CRP level was 42.82 mg/L), later on elevated transaminases (serum glutamic-oxaloacetic transaminase—174 U/L, serum glutamic-pyruvic transaminase—142 U/L), and renal involvement (elevated urea 145 mg/L and creatinine 6.7 mg/L, 24 h proteinuria: 90 mg/24 h).

Chest radiography: In the interclavicular-hilar and left subclavian areas, clustered reticular and micronodular opacities with reduced intensity were found, as well as pulmonary hila with moderately increased projection and intensity.

The diagnosis of interstitial pneumonia was established and treatment was instituted with IV drips for hydro-electrolytic rebalancing and antibiotics (Cefoperazone/sulbactam 80 mg/kg/day); treatment was continued with Prednisone 1 mg/kg/day and hydroxychloroquine sulphate 200 mg/day for the autoimmune disease.

Four days after the hospital admission, the patient showed a deterioration in his general condition: he was drowsy, he showed signs of meningeal irritation (nuchal rigidity), he no longer spoke and had swallowing disorders; Glasgow score 5.

A cranial CT scan was performed, which showed left temporo-parieto-frontal focal cerebral edema in the context of encephalitis. The repeated Mantoux test and Quantiferon assay were negative.

The repeated blood cultures were negative; infection with cytomegalovirus, Epstein Barr virus, human immunodeficiency virus, Treponema pallidum, rickettsia, and toxoplasmosis were excluded.

**Figure 8:**
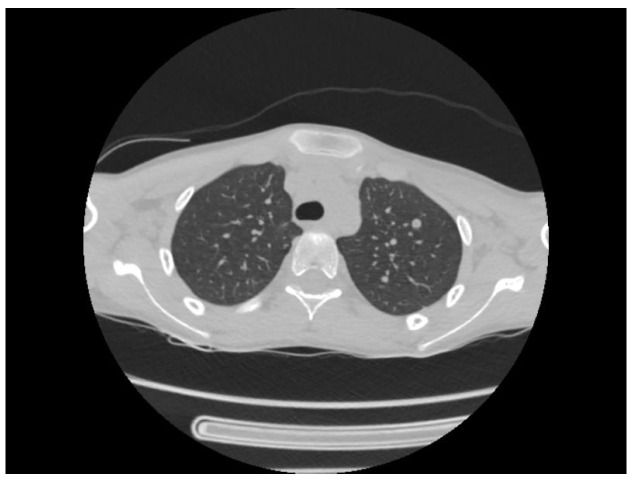
Bilateral pulmonary micronodules and iodophilic, tissular nodules. Adenopathies: in the paratraheal, paraaortic, and left interlobar areas, heterogeneous, necrotized, right hilar lymph node block extended below the carina, with microcalcifications inside; pericardial effusion.

**Figure 9:**
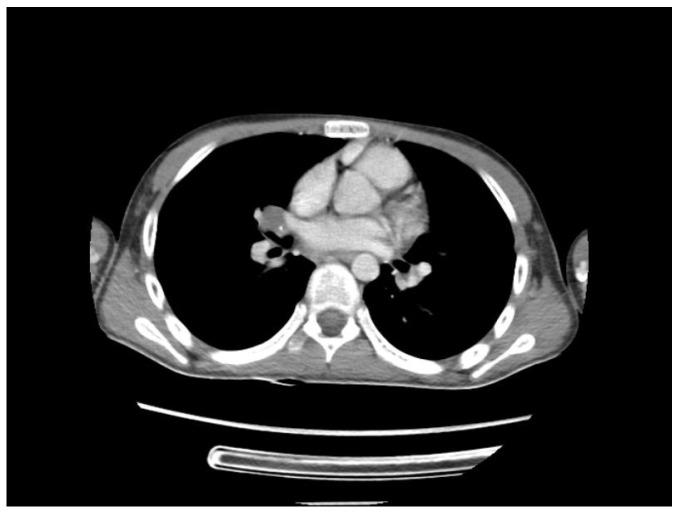
Bilateral pulmonary micronodules and iodophilic, tissular nodules. Adenopathies: in the paratraheal, paraaortic, and left interlobar areas, heterogeneous, necrotized, right hilar lymph node block extended below the carina, with microcalcifications inside; pericardial effusion.

**Figure 10:**
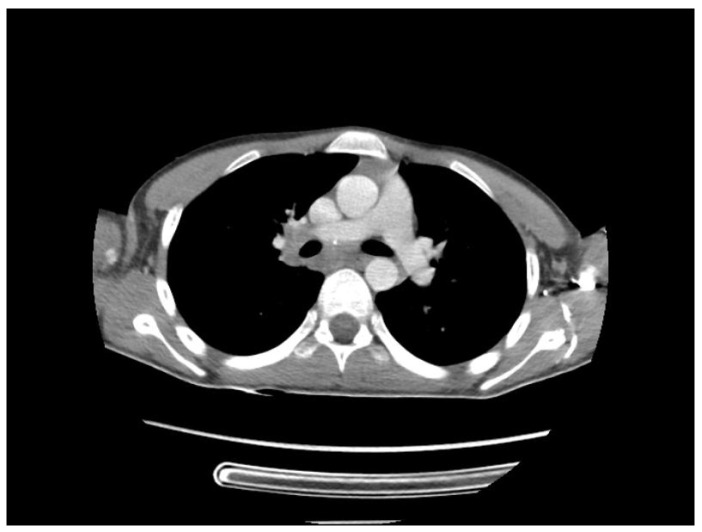
Bilateral pulmonary micronodules and iodophilic, tissular nodules. Adenopathies: in the paratraheal, paraaortic, and left interlobar areas, heterogeneous, necrotized, right hilar lymph node block extended below the carina, with microcalcifications inside; pericardial effusion.

**Figure 11:**
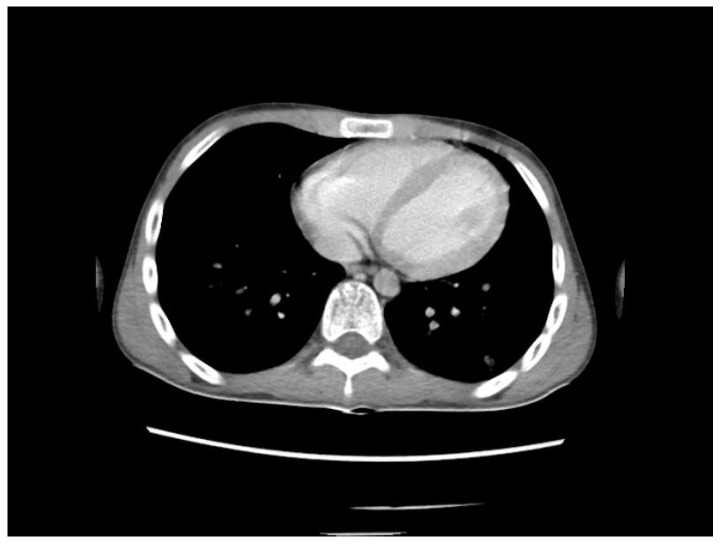
Bilateral pulmonary micronodules and iodophilic, tissular nodules. Adenopathies: in the paratraheal, paraaortic, and left interlobar areas, heterogeneous, necrotized, right hilar lymph node block extended below the carina, with microcalcifications inside; pericardial effusion.

**Figure 12:**
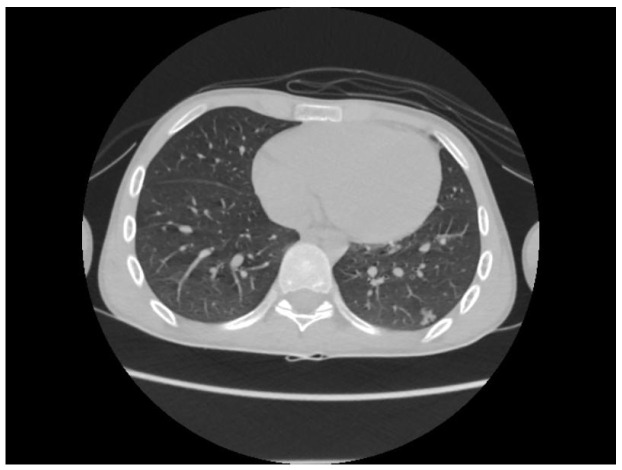
Bilateral pulmonary micronodules and iodophilic, tissular nodules. Adenopathies: in the paratraheal, paraaortic, and left interlobar areas, heterogeneous, necrotized, right hilar lymph node block extended below the carina, with microcalcifications inside; pericardial effusion.

A chest CT scan was performed, which showed bilateral pulmonary micronodules and iodophilic, tissular nodules. Adenopathies: in the paratraheal, paraaortic, and left interlobar areas, a heterogeneous, necrotized, right hilar lymph node block extended below the carina, with microcalcifications inside; pericardial effusion (Figure 8, Figure 9, Figure 10, Figure 11 and Figure 12).

The treatment regimen was changed—meropenem antibiotic 60 mg/kg/day combined with vancomycin 45 mg/kg/day, antiviral treatment, treatment to reduce cerebral edema (mannitol 1 g/kg—5 days, dexamethasone, 0.5 mg/kg/day IV drip—14 days). Because he had swallowing disorders, a nasogastric feeding tube was used for 2 days.

Considering the lack of an etiologic diagnosis, the impossibility of performing a lumbar puncture due to a cerebral edema and the fact that we could not exclude TB etiology in an immunosuppressed patient with low economic status, we initiated trial antituberculostatic therapy. The course of the patient was slowly favorable; he began to receive solid food, was no longer drowsy, used short sentences to express himself, and could walk for short distances.

The etiologic diagnosis was established on a culture medium from gastric aspirate and lumbar puncture performed 7 days after the start of treatment, when the cerebral edema decreased, and it showed clear cerebrospinal fluid, with lymphocytes present, a positive Pandy’s reaction, low glycorrhachia, and the presence of *Mycobacterium tuberculosis* bacillus (positive Barr titer) on direct examination following auramine staining. Subsequently, the culture performed on a Lowenstein–Jensen (Becton Dickinson) solid medium showed growth of *Mycobacterium tuberculosis* bacilli (BAAR-positive acid-alcohol-resistant bacilli).

The patient was transferred to the Pediatric Pneumology clinic for continuation of tuberculostatic treatment –Isoniazid, Rifampicin, Pyrazinamide and Ethambutol, daily for 3 months; subsequently, Isoniazid and Rifampicin 3 days a week, for 2 months.

Five months after the initiation of the antituberculostatic therapy, he was readmitted for SARS-CoV-2 infection, with a deteriorated general condition (septic fever, asthenia, dizziness, refusal to eat, severe headache, abdominal pain) and the Isoniazid, Rifampicin Pyrazinamide, and Ethambutol regimen was reintroduced daily, for 1 month, then Isoniazid, Rifampicin daily for 3 months, followed by Isoniazid, Rifampicin 3 days a week, for 9 months.

**Figure 13:**
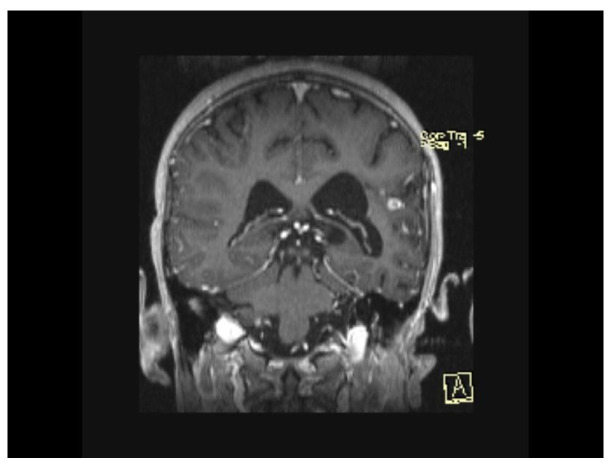
MRI Peripheral annular contrast enhancement, 9/6/6.8 mm in size, with the appearance of a tuberculoma, situated in the left temporal area. The left parietal sequelae, 4/3.8/7.1 mm and 5.1/3.7/5.3 mm in size.

**Figure 14:**
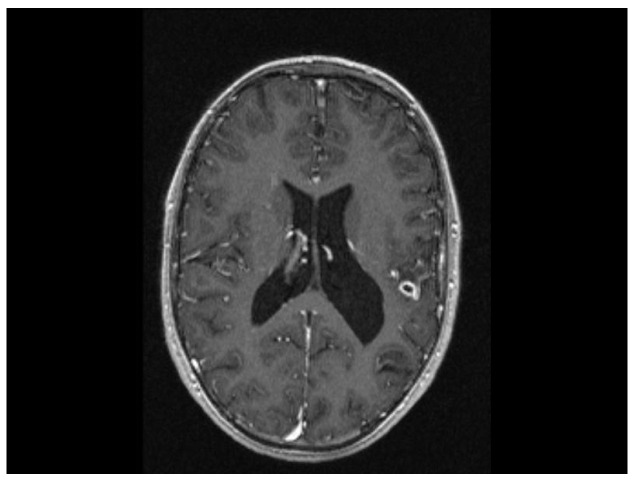
MRI Peripheral annular contrast enhancement, 9/6/6.8 mm in size, with the appearance of a tuberculoma, situated in the left temporal area. The left parietal sequelae, 4/3.8/7.1 mm and 5.1/3.7/5.3 mm in size.

**Figure 15:**
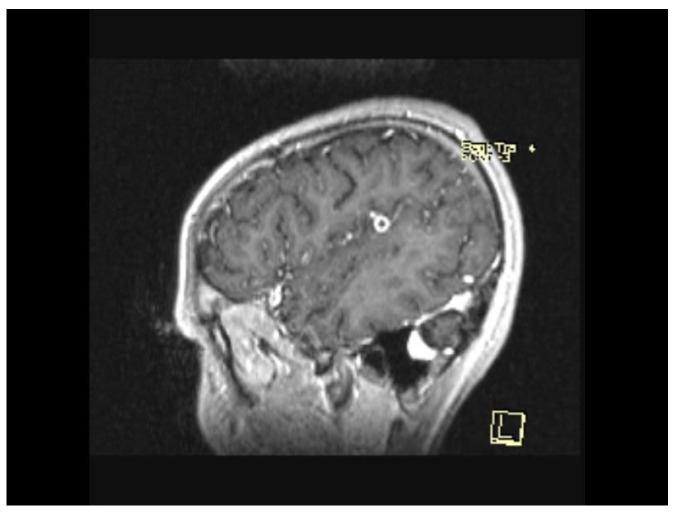
MRI Peripheral annular contrast enhancement, 9/6/6.8 mm in size, with the appearance of a tuberculoma, situated in the left temporal area. The left parietal sequelae, 4/3.8/7.1 mm and 5.1/3.7/5.3 mm in size.

The MRI scan performed 9 months after the initiation of antituberculostatic therapy showed the persistence of changes in the context of left temporo-parieto-frontal encephalitis. The non-evolving appearance of the peripheral annular contrast enhancement, 9/6/6.8 mm in size, was coupled with the appearance of a tuberculoma, situated in the left temporal area. The left parietal sequelae, 4/3.8/7.1 mm and 5.1/3.7/5.3 mm in size, were stable (Figure 13, Figure 14 and Figure 15).

Following the tuberculous meningitis, the patient did not have any symptomatic neurologic sequelae, except for a transient enuresis that remitted in 3–4 months.

**Figure 16:**
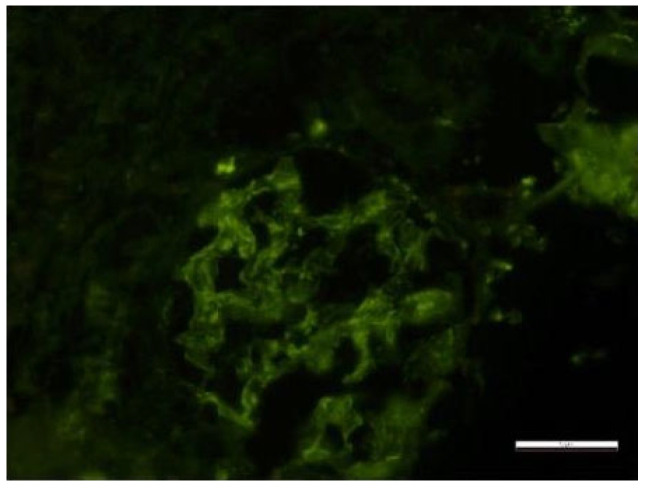
Electron microscopy Glomerulus with segmentally patent loops, with mesangial proliferation, endo- and extracapillary and cellular proliferation, with PMN in the urinary space. Rare small-medium, dense mesangial and epimembranous deposits. Activated endothelial cells, with areas of fenestrae erasure and numerous tubular-reticular structures. Segmentally erased pedicles. Lobules with endocapillary cellularity, with PMN and lymphocytes blocking the lumen, apoptotic bodies and fibrin. Fibrin in the US. Periglomerular plasma cell clusters. The content of scale bar is standard.

**Figure 17:**
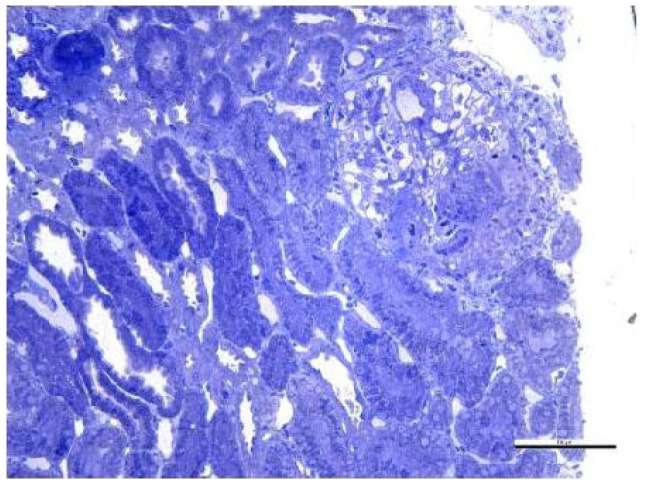
Optical microscopy. 14 Semi-fine sections stained with toluidine blue were examined. The content of scale bar is standard.

Secondary to the SARS-CoV-2 infection and because of the absence of immunosuppressive treatment during the antituberculostatic treatment, the patient later presented joint pain and renal impairment, objectified by the presence of nephritic-type proteinuria for which a renal biopsy was performed, indicating class III/V lupus nephritis with extracapillary cell proliferation (Figure 16 and Figure 17).

Fragment of renal cortex and medulla with six glomeruli. Most glomeruli with mesangial proliferation and mild mesangial matrix expansion. Three glomeruli with cellular crescents and segmental endocapillary hypercellularity. One glomerulus with significant segmental endocapillary proliferation. No interstitial fibrosis and tubular atrophy. Rare tubules with tubular epithelial cells presenting apical ballooning, with brush border loss, and with numerous lysosomes. Interlobular arteries with optical appearance within normal limits.

Therapy with mycophenolate mofetil 1 g/kg/day, indicated in the case of renal involvement, was introduced at the end of the antituberculostatic regimen; for the whole duration of therapy, the patient received only hydroxychloroquine sulphate 200 mg/day and local treatment for the skin lesions. In June 2024, we decided to initiate biological therapy with Anifrolumab 300 mg/month, with a favorable course from the articular, cutaneous and hematologic point of view.

## 3. Discussion

According to the 2023 WHO Global TB Report, almost 1.3 million children and adolescents became ill with TB in 2023, which is a strong signal of the vulnerability of this age group. Despite the decline in TB incidence in many parts of the world, between 2020 and 2023 the global incidence increased by 4.6%, reversing the downward trend observed in recent decades. In 2023, with an incidence rate of 48 cases per 100,000 inhabitants, Romania faced a worse situation than in previous years, with more than 25% of the reported TB cases in the European Union occurring in Romania [12].

The clinical onset of tuberculous meningitis is acute, subacute or in stages, but most often it is through nonspecific manifestations such as a subfebrile temperature, asthenia, sweating, headaches or weight loss [13]. Specific manifestations occur later and are represented by signs of meningeal irritation, vomiting, photophobia, gait and/or speech disorders, altered consciousness, cranial nerve palsies or neurological deficits [14].

The diagnosis is based both on a well-conducted medical history, a careful and sustained physical examination and microbiologic tests, in particular the detection of the MT bacillus in the cerebrospinal fluid by microscopy, culture, or DNA amplification techniques. Often, there is a history of exposure to an infectious case of pulmonary tuberculosis [15].

Frequently, culture and molecular tests for the *Mycobacterium tuberculosis* bacillus are negative and, even when positive, the results are rarely available within an adequate time frame so as to have a significant impact on clinical decision-making. When lumbar puncture is contraindicated, computed tomography (CT) and/or magnetic resonance imaging (MRI) of the brain or chest radiography, chest CT, or tracheobronchial or gastric aspirate are often performed to increase the possibility of establishing a correct diagnosis in a timely manner [16]. The features of intracranial tuberculosis are hydrocephalus with or without a periventricular edema, tuberculoma, infarction or pseudo-abscess [17].

A tuberculin skin test or an interferon gamma release test can help confirm sensitization to the *Mycobacterium tuberculosis* bacillus, but these tests are often falsely negative in the case of severe tuberculosis or immunosuppressed patients [16].

In our patient’s case, the initial symptoms were nonspecific, but being in an endemic area and having an immunosuppressed patient raised the suspicion of tuberculous meningitis, although the patient did not admit to contact with a person with pulmonary tuberculosis, and the tuberculin skin test result was negative. The chest radiography and cranial CT scan were non-specific, but the chest CT scan performed on the patient, due to the presence of lymph nodes blocks, strengthened the suspicion of TB meningitis and we decided to introduce antituberculostatic treatment in the absence of clear bacteriologic confirmation, in order to prevent the patient’s death or disability. The course was slowly favorable and the patient responded to the antituberculostatic treatment.

The superimposition of SARS-CoV-2 infection in our patient during the antituberculostatic treatment and the reduced treatment for SLE led to an exacerbation of SLE manifestations, in particular to the appearance of renal impairment and exacerbation of skin manifestations. This was confirmed by several studies [18,19,20] which have shown that many of these patients had more difficulty accessing healthcare during the pandemic, and that everything was compounded by the psychosocial stressors of the pandemic. On the other hand, TB, through the production of Toll-like receptor ligands, can amplify the autoimmune response and the adjustment of immunosuppressive treatment in SLE, due to the antituberculostatic therapy, leading to a worsening of the symptoms of SLE infection [21,22].

The association between the similar pathogenetic mechanisms of lupus and TB meningitis represented by complement and immunoglobulin deficiency, immunosuppression and reduced complement expression lead to a poor prognosis with high mortality [23].

There are few data in the literature regarding the association between lupus and TB meningitis in children. Thus, Cesar Adrián Martinez-Longoria reports the presence of co-infection between *Mycobacterium tuberculosis* and *Cryptococcus neoformans* in a 9-year-old girl known to have lupus. In this case, although the PPD test was negative and the chest X-ray was non-specific, the patient had a positive family history of tuberculosis, which facilitated the diagnosis [24].

Macauley P. reported in 2018 the difficulties encountered in establishing the diagnosis of tuberculous meningitis in a young woman with lupus erythematosus. Similar to our case, tuberculostatic treatment was started empirically, before confirmation by cerebrospinal fluid culture, due to the lack of response to conventional treatment for bacterial meningitis [25].

There are few data about the biological impact of SARS-CoV-2 on TB pathogenesis or the impact of COVID-19 public health control measures on TB patients [26].

Prevention, early diagnosis, and treatment of TBM and other autoimmune disorders in children is critical to reduce death and lifelong disability which represents a significant economic and social burden for families, communities, and health services [27,28].

This situation, in which the patient with SLE and TB meningitis came to the Pediatric service, is rare; thus, it was an additional reason why establishing the diagnosis was challenging and required multidisciplinary assessment (pediatrician, dermatologist, pediatric pulmonologist, pediatric nephrologist, rheumatologist, nutritionist, infection specialist, pathologist) [29,30].

In summary, our study suggests that in endemic areas and especially in patients with immunological deficit (SLE), healthcare personnel should have a high index of suspicion with regard to TB infection.

## 4. Conclusions

Tuberculous meningitis is a highly lethal, often underdiagnosed disease with nonspecific clinical and imaging manifestations, which can have a favorable outcome if the diagnosis is established early on and treatment is started promptly.

## Data Availability

The original contributions presented in this study are included in the article. Further inquiries can be directed to the corresponding author(s).

## Abbreviations

ANA	antinuclear antibodies
BMI	body mass index
CT	computed tomography
COVID-19	coronavirus disease 2019
CRP	C-reactive protein
dsDNA	double-stranded deoxyribonucleic acid
ESR	erythrocyte sedimentation rate
HIV	human immunodeficiency virus
Ig G	immunoglobulin G
Ig M	immunoglobulin M
MRI	magnetic resonance imaging
MT	*Mycobacterium tuberculosis*
WBC	white blood cell
PLT	platelet
SARS-CoV-2	severe acute respiratory syndrome coronavirus 2
SLE	systemic lupus erythematosus
TB	tuberculosis
TBM	tuberculous meningitis
